# Genetic lesioning of histamine neurons increases sleep–wake fragmentation and reveals their contribution to modafinil-induced wakefulness

**DOI:** 10.1093/sleep/zsz031

**Published:** 2019-02-05

**Authors:** Xiao Yu, Ying Ma, Edward C Harding, Raquel Yustos, Alexei L Vyssotski, Nicholas P Franks, William Wisden

**Affiliations:** 1 Department of Life Sciences, Imperial College London, UK; 2 Institute of Neuroinformatics, University of Zürich/ETH Zürich, Zürich, Switzerland; 3 UK Dementia Research Institute at Imperial College London, UK

**Keywords:** histamine, histidine decarboxylase, modafinil, tuberomammillary nucleus, caspase, lesioning, chemogenetics, wakefulness, NREM sleep

## Abstract

Acute chemogenetic inhibition of histamine (HA) neurons in adult mice induced nonrapid eye movement (NREM) sleep with an increased delta power. By contrast, selective genetic lesioning of HA neurons with caspase in adult mice exhibited a normal sleep–wake cycle overall, except at the diurnal start of the lights-off period, when they remained sleepier. The amount of time spent in NREM sleep and in the wake state in mice with lesioned HA neurons was unchanged over 24 hr, but the sleep–wake cycle was more fragmented. Both the delayed increase in wakefulness at the start of the night and the sleep–wake fragmentation are similar phenotypes to histidine decarboxylase knockout mice, which cannot synthesize HA. Chronic loss of HA neurons did not affect sleep homeostasis after sleep deprivation. However, the chronic loss of HA neurons or chemogenetic inhibition of HA neurons did notably reduce the ability of the wake-promoting compound modafinil to sustain wakefulness. Thus, part of modafinil’s wake-promoting actions arise through the HA system.

**Statement of Significance**

Different ways of manipulating histamine neurons in mice, short term with chemogenetics and long term with selective lesioning, give different effects. Short-term inhibition of histamine neurons produces nonrapid eye movement sleep. The long-term effects of killing them, on the other hand, produces a milder phenotype, with increased sleep–wake fragmentation. Both approaches underline the importance of the histamine system for generating arousal. The mice with lesions or acute inhibition of histamine neurons allowed us to test the site of action of modafinil, a popular drug to boost wakefulness and cognition. But the places where modafinil operates in the brain have proven difficult to localize. We show that modafinil requires histamine neurons for part of its wake-promoting effect.

## Introduction

The neuromodulator histamine (HA), whose neurons are located in the tuberomammillary nucleus (TMN) of the posterior hypothalamus, promotes wakefulness [1–6]. This statement is supported by extensive evidence: HA neurons are selectively wake-active [7, 8]; HA levels positively correlate with wakefulness [9]; H1 receptor inverse agonists promote nonrapid eye movement (NREM) sleep [10–12]; GABA_A_-receptor-positive allosteric modulators, such as the sleeping medication zolpidem, increase inhibition onto HA neurons and reduce the latency to NREM sleep [13]; optogenetic activation of GABAergic axons from the preoptic area in the TMN induces NREM sleep [14]; and acute optogenetic silencing of HA neurons also induces NREM sleep [15]. Conversely, acute chemogenetic stimulation of HA neurons produces increased movement and arousal [16]. Similarly, H3 receptor inverse agonists (e.g. pitolisant) increase wakefulness and promote insomnia [17, 18] by acutely driving up brain levels of HA and other amines. This effect can be used clinically to counteract daytime sleepiness in narcolepsy and other hypersomnia disorders [19].

Another compound that enhances wakefulness, but whose mechanism of action is less clear, is modafinil. Modafinil, as first recognized by Jouvet and colleagues [20, 21], is an intensively wake-promoting substance with surprisingly few adverse effects [22]. Under controlled laboratory conditions, for example, human subjects given modafinil can stay continuously awake for 2 days and one night (40 hr) [23]. The drug is approved by the US Food and Drug Administration for counteracting daytime sleepiness during narcolepsy [22, 24], shift-work sleep disorder, and obstructive sleep apnea/hypopnea syndrome [22]. Modafinil is sometimes used by service personnel, and unofficially taken (e.g. by students) for cognitive enhancement [25].

We still do not have a full understanding of how modafinil promotes wakefulness. Because modafinil cannot promote wakefulness in dopamine transporter (DAT) knockout (ko) mice [26], this seems convincing evidence that the dopamine transporter (DAT) [26–28] is critical for modafinil’s action. Modafinil antagonizes the DAT transporter and promotes a rise in dopamine levels in the basal ganglia and noradrenaline levels in the prefrontal cortex [27]. Mice with disruptions of their D1 and D2 receptor genes are insensitive to modafinil [29]. Further evidence that modafinil requires dopamine for its effects comes from humans who are homozygotes for a version of the gene-encoding catechol-O-methyltransferase that is less effective in degrading dopamine [23]. In these subjects, modafinil does not enhance wakefulness, presumably because these subjects already have high levels of dopamine [23]. Additionally, modafinil increases serotonin and HA levels in the neocortex [22] and HA levels in the anterior hypothalamus [30]. However, with all these changes, and even if the initial changes are produced by modafinil acting at the dopamine transporter, there is a complicated circle of cause and effect, and it is still unclear how modafinil works at the circuit level [27].

As seen by c-Fos expression, modafinil causes widespread excitation throughout the rodent brain, although certain nuclei such as the preoptic hypothalamic area do not show increases in c-Fos expression [31]. In cats, modafinil, immediately after administration, produced c-Fos expression mainly restricted to the anterior hypothalamus, with little expression anywhere else in the brain [32]. A few attempts at lesioning discrete brain regions have been used to try and locate a specific brain nucleus involved in modafinil’s actions. From this approach, modafinil does not appear to work by counteracting the sleep-promoting circuitry of the preoptic hypothalamus. Lesions of the ventrolateral preoptic area, whilst increasing wakefulness, have no effect on modafinil’s ability to further promote wakefulness [33]. It was also suggested that modafinil acts through the nucleus accumbens core [34]. Nucleus accumbens core lesions substantially increased the amount of time mice were awake, but modafinil did not increase wakefulness above this higher baseline level [34]. However, it could well be that capacity for arousal was already saturated in these accumbens-lesioned mice, so it is unclear if modafinil could have increased it further.

Giving modafinil systemically to mice excites orexin and HA neurons as evidenced by c-Fos expression [35, 36]. Thus, there is a possibility that some of modafinil’s effects are through the HA or orexin system, in addition to the dopamine system. Lesions of orexin neurons, however, actually increased the sensitivity of mice to modafinil [31]. This still leaves open the possibility of HA’s involvement. However, low doses of modafinil can still promote wakefulness in mice with no HA production (i.e. *hdc* ko mice) [37], suggesting no direct involvement by HA. Nevertheless, some HA neurons also corelease GABA and possibly dopamine [16, 38]. Thus, the *hdc* ko mice will leave still functional “histamine” neurons that could release other substances.

In this paper, we use mice with specific genetic lesions of their HA neurons to examine first how this lesion affects base-line (chronic) sleep–wake behavior, and second how it affects modafinil’s arousal-promoting abilities.

## Methods

### Mice

Experiments were performed in accordance with the UK Home Office Animal Procedures Act (1986); all procedures were approved by the Imperial College Ethical Review Committee. The mouse line used, *HDC-ires-Cre* (JAX labs Stock 021198), predominantly a *C57/BL6J* background was generated in our laboratory and described previously [39]. All mice used in the experiments were adult male. Mice were maintained on a reversed 12:12 hr light:dark cycle at constant temperature and humidity with ad libitum food and water.

### Adeno-associated virus transgene plasmids

Plasmid *pAAV-hSyn-DIO-hM4Di-mCherry* and *pAAV-hSyn-DIO-mCherry* was a gift from Bryan L. Roth (Addgene plasmid 44362 and 50459) [40]. Plasmid *pAAV-DIO-taCasp3-TEVp* was a gift from Nirao Shah and Jim Wells (Addgene plasmid 45580) [41]. Both transgenes have a double-floxed reading frame in an inverted orientation (“DIO”) and therefore can only be activated by Cre recombinase.

### Adeno-associated virus preparation, stereotaxic injections, and implantation of EEG/EMG electrodes

To produce AAV1/2, the adenovirus helper plasmid *pF∆6* and the adeno-associated virus (AAV) helper plasmids *pH21* (AAV1), *pRVI* (AAV2), and the *pAAV* transgene plasmids (*pAAV-hSyn-DIO-hM4Di-mCherry*, *pAAV-hSyn-DIO-mCherry*, or *pAAV-DIO-taCasp3-TEVp*) were co-transfected into HEK293 cells and the subsequent AAV particles harvested on heparin columns, as described previously [42]. Virus was bilaterally injected into the brain at a rate of 10 nL per 1 min, 100 nL for each side for the TMN injections. The AAV was injected through stainless-steel needles with Hamilton microliter #701 10 μL syringes. The injection coordinates were TMN: ML (–0.92 mm), AP (–2.70 mm), DV (–5.34 mm); ML (0.92 mm), AP (–2.70 mm), DV (–5.34 mm). After injection, the cannula was left at the injection site for 5 min and then pulled out. After injections, mice were implanted with three gold-plated miniature screw electrodes (−1.5 mm Bregma, +1.5 mm midline; +1.5 mm Bregma, −1.5 mm midline; −1 mm Lambda, 0 mm midline—reference electrode) with two EMG wires (AS634, Cooner Wire, CA) inserted into the neck The platform for the Neurologger was affixed to the skull with Orthodontic Resin power and Orthodontic resin liquid (Tocdental, UK). Mice were allowed at least 4 weeks for recovery after the surgery.

### EEG analysis and sleep–wake behavior

Two days before the recording, mice were attached with mock Neurologgers and then fitted with Neurologger 2A devices [43]. Two electroencephalograph (EEG) and two electromyograph (EMG) channels for each mouse were recorded. Spike2 (version 7.10) was used to analyze the sleep (EEG/EMG) data. The sampling rate was set up to 200 Hz. EMG was filtered by band pass between 5 and 45 Hz. EEG frequency was high-pass filtered at 0.5 Hz. The sleep states (wake, W; nonrapid eye movement, NREM, N; rapid eye movement, REM, R) were scored automatically and manually corrected. For the power spectrum analysis of control and *HDC-Cas*p3 mice, delta power (0.5–4 Hz) or theta power (4–8 Hz) was calculated during wakefulness, NREM sleep or REM sleep, respectively, during the 12 hr “lights on” (the “sleep” period) or 12 hr “lights off” (the “wake” period). To analyze the EEG power spectrum for NREM sleep, NREM sleep was assessed for 1 hr beginning with the first NREM bout that occurred after CNO injection, modafinil injection, or sleep deprivation. The fast Fourier transform (FFT) size for the power analysis was 512. EEG power was normalized to total power.

#### Locomotion activity: open-field assay

All experiments were performed during the “lights off” (active phase). The locomotion activity was detected in an activity test chamber (Med Associates, Inc.) with an ANY-maze video tracking system (Stoelting Co., United States) using a camera (FUJIFILM co).

### Behavioral protocols and drug treatments

Clozapine-N-oxide (C0832, Sigma-Aldrich, dissolved in saline, 1 mg/kg) or saline was administered (i.p.) 30 min before the start of the behavioral observations (locomotion). METHOCEL A15C Methylcellulose (00053933, Dow, United States) was dissolved in saline (0.25%). Modafinil (1811, TOCRIS, dissolved in methylcellulose/saline, 100 mg/kg) or vehicle (methylcellulose/saline) was administered i.p. For the chemogenetic experiments combined with modafinil, saline/vehicle, saline/modafinil, CNO/vehicle, or CNO/modafinil were injected at the same time at the start of “lights on” sleep period of *HDC-hM4Di* mice.

### Immunohistochemistry

Mice were anesthetized and transcardially perfused with 4% paraformaldehyde (Thermo scientific) in phosphate buffered saline (PBS) (Sigma). Brains were removed and 35 μm thick coronal sections were cut. Free-floating sections were washed in PBS three times for 5 min, permeabilized in PBS plus 0.4% Triton X-100 for 30 min, blocked by incubation in PBS plus 5% normal goat serum (NGS) (Vector), 0.2% Triton X-100 for 1 hr, and incubated with primary antibody diluted in PBS plus 2% NGS overnight at 4°C in a shaker. Incubated slices were washed three times in PBS for 10 min and incubated for 2 hr with a 1:1000 dilution of a secondary antibody (Molecular Probes) in PBS and subsequently washed three times in PBS for 10 min (all at room temperature). Primary antibodies used were rat monoclonal mCherry (1:2000, Invitrogen) and rabbit polyclonal histidine decarboxylase (HDC) (1:1000, PROGEN Biotechnik GmbH). Secondary antibodies were Alexa Fluor 488 goat anti-rabbit IgG and Alexa Fluor 594 goat anti-rat IgG (1:1000, Invitrogen Molecular Probes, United Kingdom). Slices were mounted on slides, embedded in Mowiol mounting medium (with DAPI), cover-slipped, and analyzed using a Zeiss LSM 510 inverted confocal microscope (Facility for Imaging by Light Microscopy, FILM, Imperial College). Images were acquired using Z-scan.

### Quantification and statistics

All statistical tests were performed in “Origin 2015” (Origin Lab). We used the Kolmogorov–Smirnov test for normality. We did not use statistical methods to predetermine sample sizes but our sample sizes are similar to those reported in previous publications. The individual tests are given in the figure legends. All data are given as mean ± SEM and stated in the figure legends. The data met the assumptions of the statistical tests used. All *t*-tests were two-tailed. We provided the *t* values for *t*-tests and the *F* values for ANOVA and the *p* value in the figure legends. *HDC-Cre* mice were assigned randomly to the experimental and control groups. *HDC-Cre* mice received saline, CNO, vehicle, or modafinil injections in random order. All experimental data analysis was blinded, including the analysis of EEG data and animal behavior.

## Results

### Selective chemogenetic inhibition of HA neurons promotes NREM sleep

**Figure 1.**
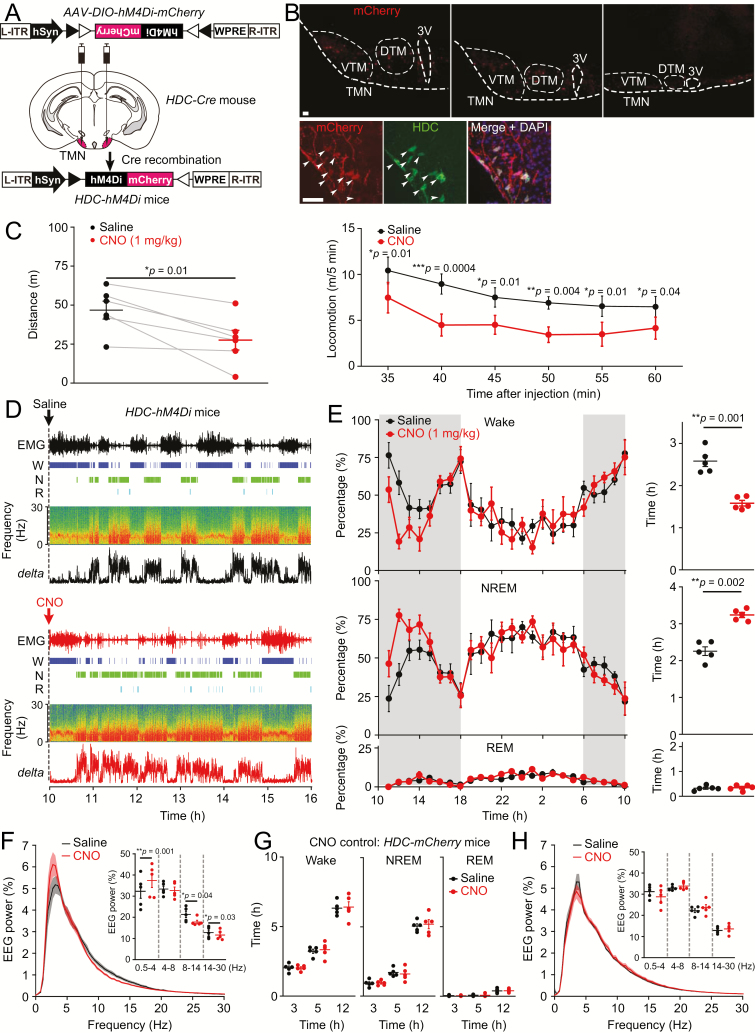
Chemogenetic inhibition of HA neurons induces sedation. (A) *AAV-DIO-hM4Di-mCherry* was injected bilaterally into the TMN area of *HDC-ires-Cre* mice to generate *HDC-hM4Di* mice. (B) Double-label immunohistochemistry from a series of coronal sections of the TMN area from an *HDC-hM4Di* mouse: mCherry (Red) and HDC (Green) confirm expression of the hM4Di-mCherry receptor in HA cells. Arrowheads indicate examples of double-labeled cells. The DAPI staining (purple) labels all the nuclei of cells in the section, indicating that most cells in the TMN are not HDC-positive. The hM4Di-mCherry receptor is extensively transported into the axons of HDC cells. Scale bars, 100 μm. VTM = the ventral part of the tuberomammillary nucleus; DTM = the dorsal part of the tuberomammillary nucleus; 3V = 3rd ventricle. (C) CNO given to *HDC-hM4Di* mice reduced locomotion. CNO was given midway through the “lights-off” active period. Distance traveled in total 30 min and locomotion speed of *HDC-hM4Di* mice that received saline (*n* = 6 mice) or 1 mg/kg CNO (*n* = 6 mice) i.p. injections. (Distance traveled: *t*(5) = 3.7, paired *t*-test, *p* = 0.013; locomotion speed: repeated measures two-way ANOVA and Bonferroni–Holm post hoc test. *F*(1, 5) = 13.496; 5 min: *t*(25) = 2.69, *p* = 0.01; 10 min: *t*(25) = 4.06, *p* = 0.0004; 15 min: *t*(25) = 2.71, *p* = 0.01; 20 min: *t*(25) = 3.15, *p* = 0.004; 25 min: *t*(25) = 2.78, *p* = 0.01; 30 min: *t*(25) = 2.11, *p* = 0.04. (D) CNO given to *HDC-hM4Di* mice evoked NREM sleep. CNO was given midway through the “lights-off” active period. An individual example of EMG, wake (W), NREM sleep (N), and REM (R) sleep, and EEG delta power spectrum of *HDC-hM4Di* mice that received saline or 1 mg/kg CNO i.p. injection. (E) CNO given to *HDC-hM4Di* mice evokes NREM sleep. The graph on the left shows the percentage and the graph on the right the total time (5 hr) of wake, NREM, and REM sleep of *HDC-hM4Di* mice that had received saline (*n* = 5 mice) or CNO (*n* = 5 mice) injections. [Paired *t*-test. Wake: *t*(4) = 7.28, *p* = 0.0018; NREM: *t*(4) = −6.84, *p* = 0.002; REM: *t*(4) = −1.74, *p* = 0.155]. Shading indicates “lights off.” (F) CNO given to *HDC-hM4Di* mice increases NREM delta power and decreases higher frequency powers. EEG power spectrum and power of different frequencies of NREM sleep of *HDC-hM4Di* mice that received saline or 1 mg/kg CNO i.p. injection. [Paired *t*-test. 0.5–4 Hz: *t*(4) = −7.61, *p* = 0.001; 4–8 Hz: *t*(4) = 0.45, *p* = 0.67; 8–14 Hz: *t*(4) = 2.92, *p* = 0.04; 14–30 Hz: *t*(4) = 3.22, *p* = 0.03]. (G) *AAV-DIO-mCherry* was injected bilaterally into the TMN area of *HDC-Cre* mice to generate *HDC-hM4Di* mice. CNO given to *HDC-mCherry* mice did not change total time (3, 5, or 12 hr) of wake, NREM, and REM sleep compared with saline injection. [Repeated measures two-way ANOVA and Bonferroni–Holm post hoc test. Wake: *F*(1, 4) = 0.066. 3 hr: *t*(8) = 0.04, *p* = 0.96; 5 hr: *t*(8) = 0.39, *p* = 0.7; 12 hr: *t*(8) = 2.71, *p* = 0.78; NREM: *F*(1, 4) = 0.007. 3 hr: *t*(8) = 0.08, *p* = 0.93; 5 hr: *t*(8) = 0.44, *p* = 0.66; 12 hr: *t*(8) = 0.58, *p* = 0.57; REM: *F*(1, 4) = 0.0006. 3 hr: *t*(8) = 0.15, *p* = 0.87; 5 hr: *t*(8) = 0.08, *p* = 0.93; 12 hr: *t*(8) = 0.19, *p* = 0.84.] All error bars represent the sem. (H) CNO given to *HDC-mCherry* mice did not affect NREM delta power and higher frequency powers. EEG power spectrum and power of different frequencies of NREM sleep of *HDC-mCherry* mice that received saline or 1 mg/kg CNO i.p. injection. [Paired *t*-test. 0.5–4 Hz: *t*(4) = 1.64, *p* = 0.17; 4–8 Hz: *t*(4) = −0.62, *p* = 0.56; 8–14 Hz: *t*(4) = −1.31, *p* = 0.25; 14–30 Hz: *t*(4) = −1.06, *p* = 0.34.]

Previously we found that acute chemogenetic (metabotropic) activation of HA neurons promoted arousal [16]. Here we tested how selective acute chemogenetic-metabotropic inhibition of those neurons influenced sleep–wake states. For metabotropic inhibition of HA neurons, we bilaterally injected *AAV-DIO-hM4Di-mCherry* into the TMN area of *HDC-ires-Cre* mice to generate *HDC-hM4Di* mice (Figure 1A). The *hM4Di-mCherry* expressed in the TMN area, including ventral part of the VTA (VTM) and dorsal part of the TMN (DTM) (Figure 1B). The Cre recombinase ensures that the expression of the cassette encoding the *hM4Di-mCherry* receptor is restricted to HA neurons. We confirmed this by double-staining sections from the TMN area using antisera against mCherry (which detects the hM4Di-mCherry receptor) and HDC, the unique marker of HA neurons (Figure 1B). The hM4Di-mCherry receptor was found abundantly expressed on axons and processes, as well as the soma of HDC-positive neurons (Figure 1B).

We then examined the consequences of chemogenetic inhibition of HA neurons at the behavioral level. CNO (1 mg/kg)-injected or saline-injected *HDC-hM4Di* mice (the same group of mice) were put into an open field to test for overt sedation. After CNO injection, *HDC-hM4Di* mice traveled less distance (46 ± 5 vs. 27 ± 6 m, *p* = 0.01) and more slowly compared with saline-injected mice (Figure 1C). We then assessed how selectively inhibiting HA neurons influenced wakefulness and sleep using EEG/EMG analysis. For *HDC-hM4Di* mice, saline or CNO (1 mg/kg) was given in the middle of the “lights on” active period. CNO administration to these mice (same group of mice) during their active period significantly increased NREM sleep for about 5 hr compared with saline injected mice (2.2 ± 0.1 vs. 3.2 ± 0.06 hr, *p* = 0.02) (Figure 1, D and E); however, the amount of REM sleep was the same between the two groups (1.0 ± 0.01 vs. 1.1 ± 0.06 hr, *p* = 0.15) (Figure 1E). We further looked at the EEG power spectrum. CNO administration to *HDC-hM4Di* mice substantially increased EEG delta power (0.5–4 Hz) (32 ± 3.3% vs. 37 ± 3.34%, *p* = 0.001) and decreased EEG power of higher frequencies (8–30 Hz) (8–14 Hz: 21 ± 1.47% vs. 17 ± 0.82%, *p* = 0.04; 14–30 Hz: 12 ± 1.16% vs. 11 ± 1.01%, *p* = 0.03) of NREM sleep compared with saline injections (Figure 1F).

To examine the specificity of CNO’s actions, we injected *AAV-DIO-mCherry* into the TMN of *HDC-Cre* mice. CNO injection into these *HDC-mCherry* mice had no effect on the amounts of sleep or wakefulness (Figure 1G) or EEG power spectrum (Figure 1H) compared with saline-injected mice. Note: we found that CNO given at higher doses of 5 or 1 mg/kg to a variety of other control mouse lines did not alter locomotion or change the amounts of sleep–wake compared with saline injections [44, 45].

### Ablation of HA neurons does not affect the overt sleep–wake cycle but induces more fragmented wakefulness and NREM sleep

**Figure 2.**
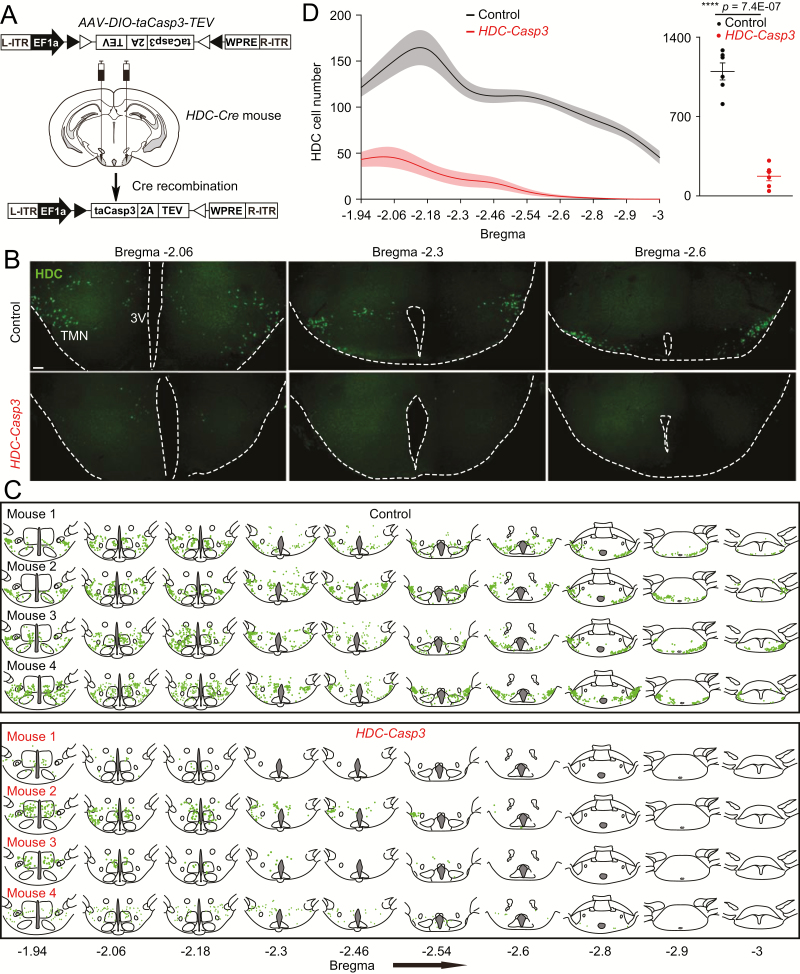
Selective genetic lesioning of HA neurons. (A) *AAV-DIO-taCasp3-TEV* was bilaterally injected into the TMN area of *HDC-ires-Cre* mice to generate *HDC-Casp3* mice. To generate the controls, *AAV-DIO-taCasp3-TEV* was injected bilaterally into the TMN area of *HDC-Cre*-negative mice. (B) Casp3 efficiently kills HDC neurons. Six weeks after the *AAV-DIO-taCasp3-TEV* injections, immunohistochemistry was undertaken for HDC. Illustrative examples of HDC immunohistochemistry from a control mouse and an *HDC-Casp3* mouse coronal section for the TMN area (three representative coronal sections on the rostral–caudal axis for HDC-immunostaining in the TMN are shown). The green dots indicate neuronal cell bodies stained for HDC, 3V, third ventricle. Scale bar, 200 μm. (C) Mapping the extent of HDC cell lesioning. Line drawings of sections showing HDC-positive cells (green dots) from individual control mice (*n* = 4 mice, designated as “mouse1” through to “mouse 4”) and *HDC-Casp3* mice (*n* = 4 mice, designated as “mouse 1” through to “mouse 4”) along most of the rostral–caudal axis of the TMN area (bregma −1.94 to bregma −3). Few HDC-positive cells remained in the sections from the *HDC-Casp3* mice. (D) Counts of HDC cell numbers along the rostral–caudal axis per section (bregma −1.94 to bregma −3) (left-hand graph) and total HDC cell numbers of control mice (*n* = 6 mice) and *HDC-Casp3* mice (*n* = 6 mice) [*t*(10) = 10.86, unpaired *t*-test, *p* = 7.4E-7] (right-hand graph). All error bars represent the sem. The shaded envelopes on left-hand graph indicate sem.

We next conducted chronic lesioning experiments to access the function of HA neurons in regulating sleep and wakefulness. To selectively lesion HA neurons, *AAV-DIO-taCasp3-TEV* was bilaterally delivered into the TMN area of adult *HDC-ires-Cre* mice to generate *HDC-Casp3* mice (Figure 2A). For the control group, *AAV-DIO-taCasp3-TEV* was injected into the TMN area of Cre-negative littermates. Six weeks after the AAV injections, the efficiency of the lesion was assessed with immunocytochemistry using an HDC antibody. Compared with AAV-injected Cre-negative control animals, the number of HDC-positive cells was substantially reduced in *HDC-Casp3* mice (Figure 2B). We mapped HDC expression in both control and *HDC-Casp3* mice throughout the entire TMN area (Figure 2, B and C). Nearly 85% of the HDC-cells were killed in *HDC-Casp3* animals (1097 ± 75 vs.173 ± 40, *p* = 7.4E-7) (Figure 2D).

**Figure 3.**
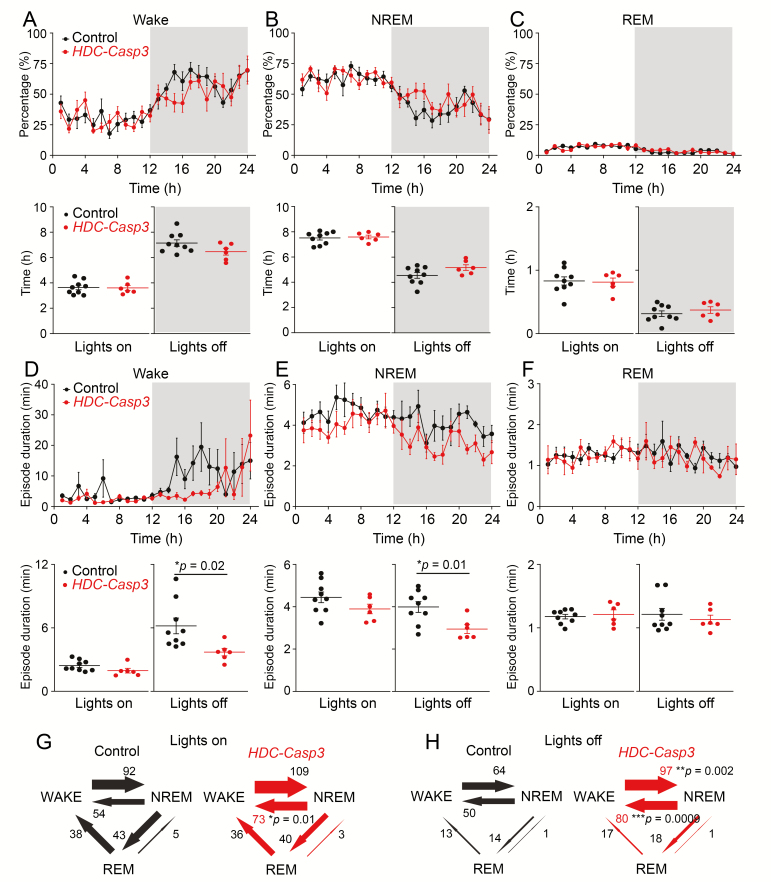
Ablation of HA neurons does not affect the overt sleep–wake cycle but induces more fragmented wakefulness and NREM sleep. (A, B, C) Percentage and time of wake, NREM, and REM sleep of *HDC-Casp3* mice (*n* = 6 mice) and control mice (*n* = 9 mice) over the 24 hr cycle. [Unpaired *t*-test. Lights on: wake *t*(13) = 0.12, *p* = 0.9; NREM *t*(13) = −0.3, *p* = 0.76; REM *t*(13) = 0.19, *p* = 0.84; lights off: wake *t*(13) = 1.76, *p* = 0.1; NREM *t*(13) = −1.85, *p* = 0.08; REM *t*(13) = −0.83, *p* = 0.41]. (D, E, F) Episode duration of wake, NREM, and REM sleep of *HDC-Casp3* mice (*n* = 6 mice) and control mice (*n* = 9 mice) across the 24 hr cycle and “lights on” and “lights off” periods [Unpaired *t*-test. Lights on: wake *t*(13) = 1.74, *p* = 0.1; NREM *t*(13) = 1.51, *p* = 0.15; REM *t*(13) = −0.46, *p* = 0.64; lights off: wake *t*(13) = 2.57, *p* = 0.02; NREM *t*(13) = 2.91, *p* = 0.01; REM *t*(13) = 0.64, *p* = 0.52.] (G, H) Vigilance state transitions of *HDC-Casp3* mice (*n* = 6 mice) and control mice (*n* = 9 mice) during the “lights on” and “lights off” periods [Unpaired *t*-test. Lights off: wake to NREM *t*(13) = −3.69, *p* = 0.002; NREM to wake *t*(13) = −4.26, *p* = 9.2E-4.] All error bars represent the sem.

We performed sleep–wake recordings of control and *HDC-Casp3* mice over the 24 hr cycle. These recordings took place 6 weeks after the *AAV-DIO-taCasp3-TEV* injections. As found for *hdc* knockout mice [46], the 24 hr spontaneous sleep–wake pattern was similar between *HDC-Casp3* mice and control mice (Figure 3, A–C). Over 24 hr, the amount of wakefulness, NREM, or REM sleep of *HDC-Casp3* mice did not differ from control littermates (AAV injected Cre-negative mice). Although the amount of wakefulness was slightly decreased and the amount of NREM sleep slightly increased in *HDC-Casp3* mice during the 12 hr “lights off” period, these changes did not reach significance (wake: 7.1 ± 0.25 vs. 6.4 ± 0.26 hr, *p* = 0.1; NREM: 4.5 ± 0.22 vs. 5.1 ± 0.22 hr, *p* = 0.08) (Figure 3, A and B). Of note, the *HDC-Casp3* mice became aroused more slowly than control mice after the start of the “lights off” period (from time 14:00 to 17:00).

**Figure 4.**
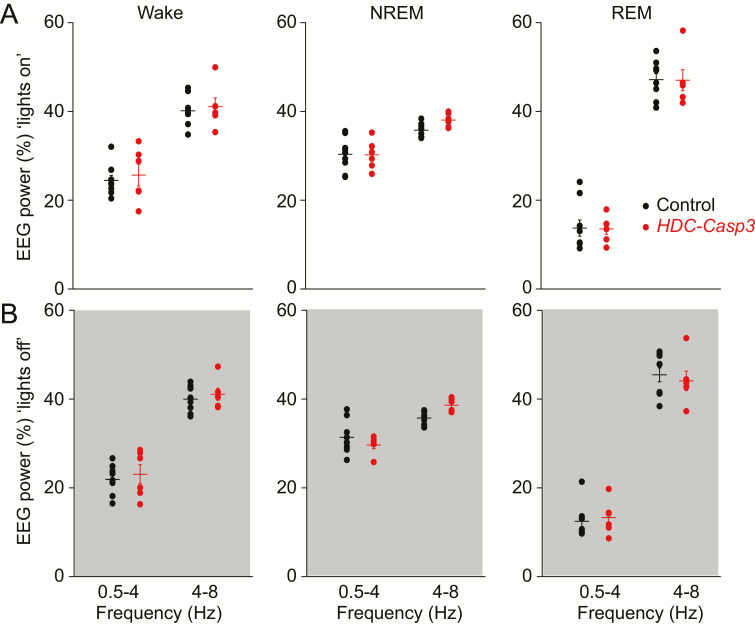
Ablation of HA neurons does not affect the EEG power spectrum during the spontaneous sleep–wake cycle. (A, B) EEG delta (0.5–4 Hz) and theta (4–8 Hz) power of wakefulness, NREM sleep, or REM sleep of control and *HDC-Casp3* mice during the 12 hr “lights on” period (A) or the 12 hr “lights off” period (B). Two-way ANOVA and Bonferroni–Holm post hoc test. (Lights on: wake *t* = 0.65, *p* = 0.51; NREM *t* = 1.06, *p* = 0.29; REM *t* = −0.08, *p* = 0.93; lights off: wake *t* = 0.84, *p* = 0.4; NREM *t* = 0.63, *p* = 0.52; REM *t* = −0.16, *p* = 0.87.)

We looked into the sleep microarchitecture of the *HDC-Casp3* mice. The episode duration of wakefulness and NREM sleep decreased in *HDC-Casp3* mice, particularly during the “lights off” active period (Figure 3, D and E) (wake: 6.18 ± 0.74 vs. 3.7 ± 0.35 min, *p* = 0.02; NREM: 3.98 ± 0.25 vs. 2.94 ± 0.2 min, *p* = 0.01). The REM sleep episode duration did not differ between *HDC-Casp3* mice and control mice (1.21 ± 0.09 vs. 1.13 ± 0.06 min, *p* = 0.52) (Figure 3F). Looking in more detail at the sleep–wake transitions, the *HDC-Casp3* mice had more NREM to wake transitions during both the “lights on” and “lights off” periods (Figure 3, F and G) (50 ± 4 vs. 80 ± 6, *p* = 0.0009) and more wake to NREM sleep transitions during the “lights off” period (Figure 2F) (64 ± 5 vs. 97 ± 7, *p* = 0.002). Finally, we assessed the EEG power spectrum of control and *HDC-Casp3* mice during each vigilance state during the 12 hr “lights on” period or 12 hr “lights off” period. Both delta (0.5–4 Hz) and theta power (4–8 Hz) of control mice did not differ from *HDC-Casp3* mice (Figure 4). The above results suggest that the loss of HA neurons has been compensated for—the mice were not overtly sleepy, there were no obvious effects on the amounts of sleep and wake in the spontaneous sleep–wake cycle, but HA neurons are needed for consolidating wakefulness, otherwise NREM sleep intrudes.

### Modafinil promotes wakefulness partially through HA neurons

**Figure 5.**
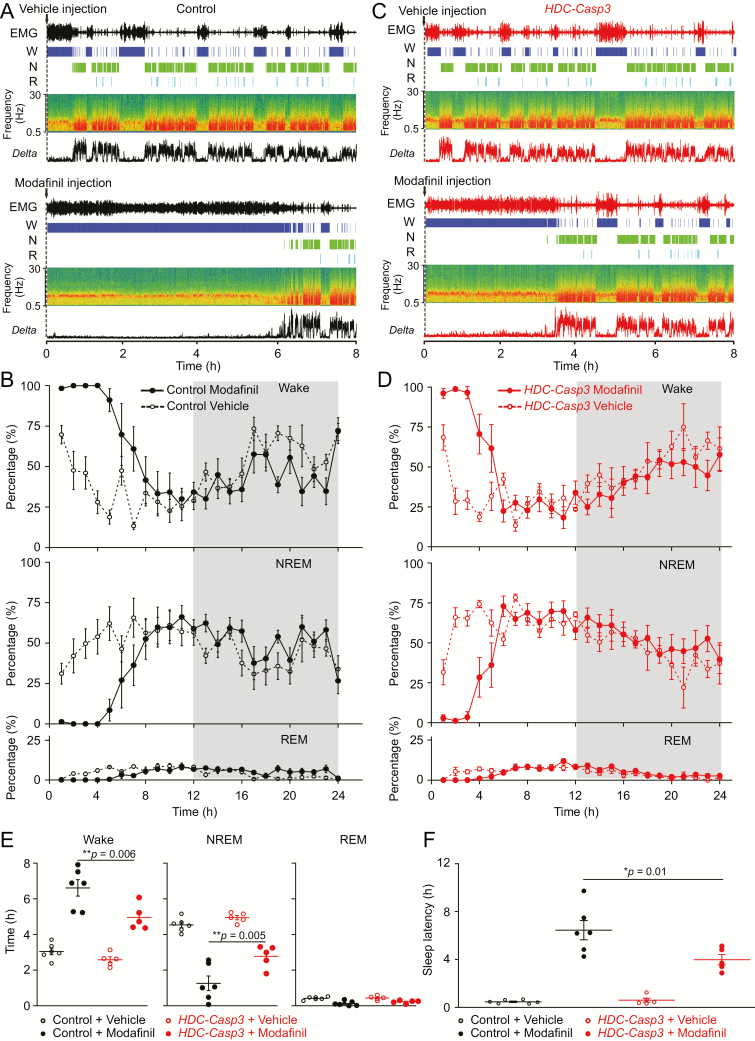
Chronic lesioning of HA neurons partially attenuates modafinil-induced wakefulness. (A) An individual example of EMG, wake (W), NREM sleep (N), and REM (R) sleep, and EEG delta power of a control mouse that received vehicle or modafinil by i.p. injection. (B) Percentages of wake, NREM, and REM sleep of control mice (*n* = 6 mice) that received vehicle or modafinil i.p. injection. (C) An individual example of EMG, wake (W), NREM sleep (N), and REM (R) sleep, and EEG delta power of an *HDC-Casp3* mouse that received vehicle or modafinil by i.p. injection. (D) Percentages of wake, NREM, and REM sleep of *HDC-Casp3* mice (*n* = 5 mice) that received vehicle or modafinil injections. (E) Time (8 hr) of wake, NREM, and REM sleep of control mice (*n* = 6 mice) and *HDC-Casp3* mice (*n* = 5 mice) that received vehicle or modafinil injections (control modafinil vs. CASP3 modafinil: wake: *F*(1, 4) = 5.57, *t*(4) = 5.1, *p* = 0.006; NREM: *F*(1, 4) = 6.48, *t*(4) = 5.42, *p* = 0.005; REM: *F*(1, 4) = 1.11, *t*(4) = 2.3, *p* = 0.08. (F) Sleep latency to NREM sleep of control mice (*n* = 6 mice) and *HDC-Casp3* mice (*n* = 5 mice) that received vehicle or modafinil injections. [*F*(1, 4) = 7.56, control modafinil vs. CASP3 modafinil: *t*(4) = 4.18, *p* = 0.01.] Repeated measures two-way ANOVA and Bonferroni–Holm post hoc test. All error bars represent the sem.

Using *HDC-Casp3* mice, we next examined whether the wake-promoting effect of modafinil depends on the HA system. We systemically gave modafinil or vehicle by i.p. injection to *HDC-Casp3* mice and control mice. Control mice were Cre-negative littermates that received AAV *AAV-DIO-taCasp3-TEV*. We then compared modafinil’s actions on wakefulness in control mice and *HDC-Casp3* mice. In control mice, consistent with previous reports, modafinil induced continuous wakefulness for about 7 hr with 100% wakefulness in the first 3 hr (Figure 5, A and B); however, in *HDC-Casp3* mice, modafinil increased wakefulness for only about 4 hr (Figure 5, C and D). During the first 8 hr, modafinil-treated *HDC-Casp3* mice had less wakefulness (6.6 ± 0.46 vs. 4.9 ± 0.3 hr, *p* = 0.006), but more NREM sleep compared with modafinil-treated control mice (Figure 5E) (NREM: 1.2 ± 0.41 vs. 2.7 ± 0.27 hr, *p* = 0.005), but REM sleep did not alter (REM: 0.1 ± 0.06 vs. 0.23 ± 0.03 hr, *p* = 0.08). After vehicle injection, the sleep latency in control mice was identical to *HDC-Casp3* mice (Figure 5F) (0.57 ± 0.07 vs. 0.66 ± 0.19 hr, *p* = 0.74); however, after modafinil injection, the sleep latency of *HDC-Casp3* mice was reduced by about half compared with control mice (6.7 ± 0.9 vs. 3.9 ± 0.4 hr, *p* = 0.01) (Figure 5F).

**Figure 6.**
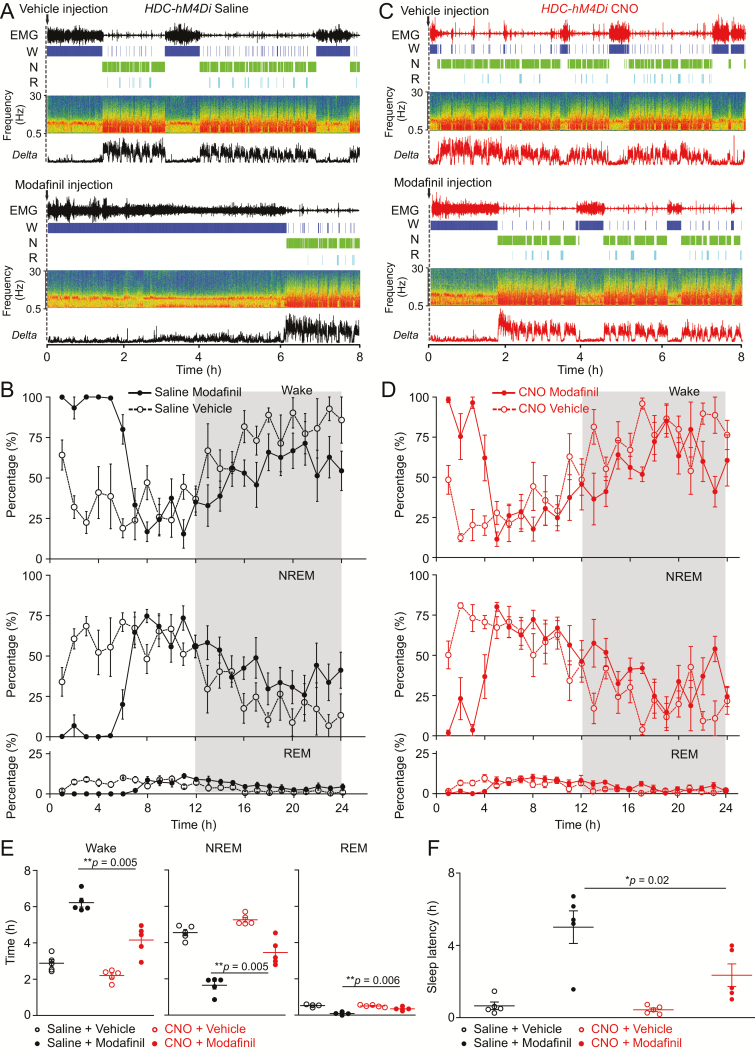
Chemogenetic inhibition of HA neurons partially attenuates modafinil-induced wakefulness. (A) An individual example of EMG, wake (W), NREM sleep (N), and REM (R) sleep, and EEG delta power of a control mouse that received saline and vehicle or saline and modafinil by i.p. injection. (B) Percentages of wake, NREM, and REM sleep of control mice (*n* = 5 mice) that received saline and vehicle or saline and modafinil i.p. injection. (C) An individual example of EMG, wake (W), NREM sleep (N), and REM (R) sleep, and EEG delta power of an *HDC-hM4Di* mouse that received CNO and vehicle or CNO and modafinil by i.p. injection. (D) Percentages of wake, NREM and REM sleep of *HDC-hM4Di* mice (*n* = 5 mice) that received CNO and vehicle or CNO and modafinil injections. (E) Time (8 hr) of wake, NREM, and REM sleep of *HDC-hM4Di* saline-injected mice (*n* = 5 mice) and *HDC-hM4Di* CNO-injected mice (*n* = 5 mice) that received vehicle or modafinil injections [saline and modafinil vs. CNO and modafinil: wake: *F*(1, 4) = 11.52, *t*(4) = 5.5, *p* = 0.005; NREM: *F*(1, 4) = 8.89, *t*(4) = 5.59, *p*=0.005; REM: *F*(1, 4) = 44.08, *t*(4) = 5.1, *p* = 0.006.] (F) Sleep latency to NREM sleep of *HDC-hM4Di* saline-injected mice (*n* = 5 mice) and *HDC-hM4Di* CNO-injected mice (*n* = 5 mice) that received vehicle or modafinil injections. [*F*(1, 4) = 10.23, saline and modafinil vs. CNO and modafinil: *t*(4) = 3.56, *p* = 0.02.] Repeated measures two-way ANOVA and Bonferroni–Holm post hoc test. All error bars represent the sem.

Using chemogenetic inhibition, we further examined whether the wake-promoting effect of modafinil depends on the HA system. Saline and modafinil, or CNO and modafinil, were injected into *HDC-hM4Di* mice. Consistent with the above results (Figure 5), modafinil induced continuous wakefulness for about 7 hr with nearly 100% wakefulness in the first 3 hr (Figure 6, A and B) in saline-injected *HDC-hM4Di* mice; however, in CNO-injected *HDC-hM4Di* mice, modafinil increased wakefulness for only about 4 hr (Figure 6, C and D). During the first 8 hr, CNO/modafinil-injected *HDC-hM4Di* mice had less wakefulness (6.2 ± 0.23 vs. 4.1 ± 0.36 hr, *p* = 0.005), but more NREM sleep compared with the saline/modafinil-treated control mice (Figure 4E) (1.6 ± 0.21 vs. 3.4 ± 0.32 hr, *p* = 0.005), and more REM sleep (0.1 ± 0.03 vs. 0.36 ± 0.04 hr, *p* = 0.006). After vehicle injection, the sleep latency in saline-injected mice was identical to CNO-injected *HDC-hM4Di* mice (Figure 4F) (0.65 ± 0.2 vs. 0.43 ± 0.09 hr, *p* = 0.78); however, after modafinil injection, the sleep latency of CNO-injected mice was reduced by about half compared with saline-injected mice (5 ± 0.9 vs. 2.34 ± 0.6 hr, *p* = 0.02) (Figure 6F). These results suggest that the wake-promoting effect of modafinil is partially due to activating the HA system.

### Ablation of HA neurons does not affect sleep homeostasis after modafinil-induced wakefulness or sleep deprivation

**Figure 7.**
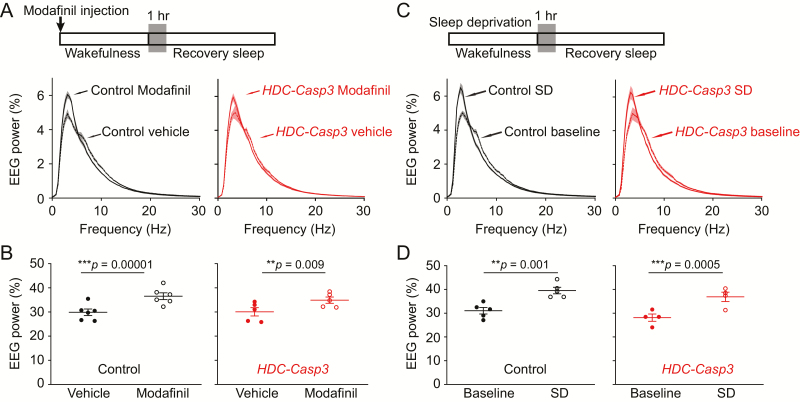
Ablation of HA neurons does not affect sleep homeostasis after sleep deprivation or modafinil-induced wakefulness. (A, B) EEG power spectrum of NREM sleep (A) and total delta power (0.5–4 Hz) of NREM sleep (B) of control and *HDC-Casp3* mice during the first hour of recovery sleep after modafinil-induced wakefulness. (C, D) EEG power spectrum of NREM sleep (A) and total delta power (0.5–4 Hz) of NREM sleep (B) of control and *HDC-Casp3* mice during the first hour of recovery sleep after sleep deprivation. [Paired *t*-test. (B) Control: *t*(5) = −17.52, *p* = 0.00001; *HDC-Casp3*: *t*(5) = −4.62, *p* = 0.009; (D) control: *t*(4) = −7.6, *p* = 0.001; *HDC-Casp3*: *t*(3) = −15.41, *p* = 0.0005.]

Finally, we tested whether HA neurons are involved in regulating sleep homeostasis after a prolonged wakefulness by examining the delta power of NREM sleep during the starting period (1 hr) of recovery sleep. We first looked at the EEG power spectrum of control and *HDC-Casp3* mice after vehicle or modafinil injection (Figure 7A). After modafinil injection, the delta power in both control mice and *HDC-Casp3* mice was increased during the first hour of recovery sleep compared with vehicle injection (Figure 7, A and B) (control: 29 ± 3% vs. 36 ± 3%, *p* = 0.00001; *HDC-Casp3*: 30 ± 3% vs. 34 ± 1%, *p* = 0.009). In addition, we performed sleep deprivation experiments to test the contribution of HA neurons to the homeostatic response within the natural sleep circuitry (Figure 7C).

Similar to the modafinil injections, both control mice and *HDC-Casp3* mice had an increased EEG delta power during the first hour of recovery NREM sleep compared with their spontaneous baseline NREM sleep (Figure 7, C and D) (control: 31 ± 1% vs. 39 ± 1%, *p* = 0.001; *HDC-Casp3*: 28 ± 1% vs. 36 ± 1%, *p* = 0.0005). These results suggest that the HA system did not participate in the regulation of sleep homeostasis.

## Discussion

Our work has shown several aspects of interest for the neuronal HA system. First, selective chemogenetic inhibition of HA neurons produced NREM sleep. But, by contrast, genetic lesioning of HA neurons chronically induced in the adult resembles the phenotype of *hdc* ko mice (i.e. mice without a functional *hdc* gene from conception) [46]. This suggests that a similar compensatory mechanism occurs in the adult as in the developing *hdc* knockout mice. Second, the HA system is not required for sleep homeostasis (defined as the increase in NREM delta power seen in the immediate part of NREM recovery sleep after sleep deprivation), as also concluded in an independent study [9]. Third, HA neurons are required for part of modafinil’s actions in promoting wakefulness.

The results obtained with the acute pharmacogenetic and optogenetic manipulations of the HA system that induce NREM sleep (see Introduction) contrast with the results of lesioning of the same cells. It is often noted that Von Economo identified flu-induced lesions in the human posterior hypothalamus, more specifically in the posterior wall of the third ventricle, as producing excessive somnolence (*encephalitis lethargica*) [47]. Hence, Von Economo suggested that the posterior hypothalamus contained a wake-promoting area [47] and so founded the modern concept that there are wake and sleep-promoting centers in the brain [1]. Ironically, this result (at least on the basis of lesioning the TMN) is not borne out in work on rodents. Killing of neurons in the rat TMN area using saporin-orexin ligand produced no effect on sleep amounts [48, 49]. Multiple cell types are lesioned in the TMN in this model, because orexin-saporin kills all neurons that express the orexin receptors, and this expression of the orexin receptor is not restricted to HA neurons, but also occurs in other TMN neurons.

A similar lack of effect of the HA system on sleep–wake was found from lesioning at the gene level. *Hdc* gene knockout mice, which lack the capability to synthesize HA, are not dramatically impaired in their sleep–wake profile [46]. The mice do have a more limited enthusiasm to investigate novel objects, possibly because they are less aroused, and they have more sleep–wake fragmentation. Additionally, *hdc* ko mice are more sleepy at the light-to-dark transition that marks the start of their active period. This lack of strong phenotype in *hdc* ko mice compared with the results obtained with acute experiments antagonizing or stimulating the HA system suggests some form of compensation in the *hdc* knockout mice, which perhaps occurs during brain development. A similar situation pertains to H1 receptor ko mice which have only mild increases in NREM sleep bouts, possibly because of an upregulated cholinergic system [50]. On the other hand, mice with a permanent upregulation of *hdc* gene expression also have sleep–wake fragmentation [51]. Yet, mice with no H3 receptors show reduced wakefulness in nonstressful situations [52], contrasting with the wake-promoting effects of H3 inverse agonists. Perhaps these results show the extraordinary unpredictability of compensatory systems (sometimes there is compensation, sometimes not, sometimes partial) in the brain.

In any case, the HA-lesioned mice in our study reproduce the sleep–wake phenotype of *hdc* knockout mice to a remarkable degree [46]. HA-lesioned mice have more sleep–wake fragmentation, less arousal at the start of lights-off, the active period of the mice. Thus, HA-lesioned mice are likely to have the same compensatory mechanism present in the *hdc* ko mice, and this effect can emerge in the adult and not only during development. Previously, we found some HA neurons corelease GABA in the neocortex [16]. In the *hdc* ko mice, these neurons can still corelease GABA, whereas in our HA-lesioned mice, both GABA and HA release will be abolished. Yet the phenotypic effects of *hdc* gene and HA cell lesions are the same. Knockdown of the vesicular GABA transporter gene expression from HA neurons produced hyperactive animals. We are not sure why we do not get a larger phenotype in HA cell-lesioned mice compared with *hdc* ko mice. Given the unpredictability of compensations, perhaps the loss of GABA signaling from HA cells has been more compensated than loss of HA signaling.

In spite of compensations at the behavioral level, lesioning studies can be useful, whether cellular or genetic, to reveal drug targets. For example, DAT, D1, and D2 knockout mice are largely insensitive to modafinil [26, 29], and H3 knockout mice are insensitive to the wake-enhancing effects of H3 inverse agonists [37, 52]. Along similar lines, our cellular lesioning and inhibitory chemogenetic studies suggest that HA neurons contribute to the mechanism of modafinil-induced wakefulness. There is, however, likely to be complex positive feedback in the way that modafinil promotes wakefulness.

VTA dopamine neurons promote wakefulness, in part, via the nucleus accumbens [53, 54]. As modafinil antagonizes DAT and raises dopamine levels, and indeed requires DAT for its wake-promoting actions, it seems that raised dopamine in the nucleus accumbens is the obvious way that modafinil promotes wakefulness. But HA neurons also express dopamine receptors and can be excited by dopamine agonists [38]; HA neurons may also be able to synthesize and release dopamine [38]. Thus, the raised dopamine levels, sensed either by the HA soma in the TMN or by HA axons in, for example, neocortex or nucleus accumbens will promote HA release, and possibly even dopamine release. HA probably promotes direct wakefulness in the neocortex via postsynaptic H1 receptors, but also HA directly stimulates the firing of dopamine neurons in the VTA and other amine neurons [18], further promoting wakefulness. Consequently, there will be a number of wake-promoting pathways operating in parallel, some involving dopamine acting through, e.g. the nucleus accumbens on D2 receptors and others where the HA neurons are probably excited by dopamine and then the released HA produces wakefulness via many targets, e.g. the basal forebrain cholinergic neurons [9] or the neocortex or nucleus accumbens.

A further point is that in *hdc* ko mice, modafinil still produces as much wakefulness as in mice with an intact *hdc* gene [37]. Possible reasons for this discrepancy are that the earlier study used a lower dose of modafinil, or that dopamine or GABA released from the HA axons in the neocortex, rather than HA itself, mediate some of the effects of modafinil. Another interpretation is that HA neurons are needed for maintaining some of modafinil’s actions, but not for initiating them. Background strains of mice could also influence the sensitivity to modafinil, as clearly there is considerable variation in the sensitivity of humans to the drug, which is likely to be based on genetic differences [23].

In conclusion, selective genetic lesioning of adult HA neurons shows their requirement for consolidating wakefulness and for sustaining some of the wake-promoting effects of modafinil. The effects on vigilance state produced by acute inhibition of HA neurons compared with those produced by their long-term removal are much larger.

## Funding

This work was supported by the Wellcome Trust (joint Investigator Award, 107839/Z/15/Z [to N.P.F.] and 107841/Z/15/Z [to W.W.]), the UK Dementia Research Institute (to N.P.F. and W.W.), and a UK-China Scholarships for Excellence/China Scholarship scheme (to Y.M.).

*Conflict of interest statement*. None declared.
