# Corrigendum: Genetic lesioning of histamine neurons increases sleep–wake fragmentation and reveals their contribution to modafinil-induced wakefulness

**DOI:** 10.1093/sleep/zsz107

**Published:** 2019-06-01

**Authors:** Xiao Yu, Ying Ma, Edward C Harding, Raquel Yustos, Alexei L Vyssotski, Nicholas P Franks, William Wisden

In the article Genetic lesioning of histamine neurons increases sleep–wake fragmentation and reveals their contribution to modafinil-induced wakefulness, *Sleep*, zsz031, https://doi.org/10.1093/sleep/zsz031, affiliation one was incorrectly listed as “Department of Life Sciences”. This has been corrected to “Department of Life Sciences, Imperial College London, UK”.

